# Combined androgen blockade achieved better oncological outcome in androgen deprivation therapy for prostate cancer: Analysis of community‐based multi‐institutional database across Japan using propensity score matching

**DOI:** 10.1002/cam4.1735

**Published:** 2018-08-27

**Authors:** Mizuki Onozawa, Hideyuki Akaza, Shiro Hinotsu, Mototsugu Oya, Osamu Ogawa, Tadaichi Kitamura, Kazuhiro Suzuki, Seiji Naito, Mikio Namiki, Kazuo Nishimura, Yoshihiko Hirao, Taiji Tsukamoto

**Affiliations:** ^1^ Department of Urology School of Medicine International University of Health and Welfare Chiba Japan; ^2^ Research Center for Advanced Science and Technology University of Tokyo Tokyo Japan; ^3^ Department of Biostatistics and Clinical Epidemiology Sapporo Medical University Sapporo Japan; ^4^ Department of Urology School of Medicine Keio University Tokyo Japan; ^5^ Department of Urology Kyoto University Graduate School of Medicine Kyoto Japan; ^6^ Department of Urology Shinsui Clinic Tokyo Japan; ^7^ Department of Urology Gunma University Graduate School of Medicine Gunma Japan; ^8^ Department of Urology Harasanshin Hospital Fukuoka Japan; ^9^ Department of Urology Hasegawa Hospital Toyama Japan; ^10^ Department of Urology Osaka International Cancer Institute Osaka Japan; ^11^ Department of Urology Osaka Gyoumeikan Hospital Osaka Japan; ^12^ Department of Urology Sapporo Medical University Sapporo Japan

**Keywords:** castration‐resistant, disease‐free survival, hormone‐sensitive, nonsteroidal anti‐androgens, propensity score, prostatic neoplasms

## Abstract

**Background:**

This study investigated how differences in the method of the first‐line androgen deprivation therapy (ADT) affected the time to castration‐resistant prostate cancer.

**Methods:**

The Japan Study Group of Prostate Cancer compiled a nationwide community‐based database on prostate cancer patients who underwent ADT. That database included 13 774 patients who were started on ADT by surgical or medical castration alone (monotherapy group, 5395 cases) or ADT in combination with a nonsteroidal anti‐androgen (combined androgen blockade (CAB) group, 8379 cases). We used logistic regression analysis with background factors as independent factors to calculate propensity scores in regard to selection of CAB. Next, for 8826 cases of propensity score‐matched patients, we compared the survival rates in the two groups.

**Results:**

The CAB group showed a significantly better progression‐free survival (PFS) rate (65.6% vs 59.6% at 5 years; median time to progression, 11.6 vs 7.1 years; hazard ratio in the CAB group: 0.78, with a 95% confidence interval of 0.72 to 0.84; *P* < 0.001). In subgroup analysis based on the background factors, the PFS rate was generally better in the CAB group in all risk subgroups except for those having significant risk factors.

**Conclusion:**

Propensity score matching analysis revealed the prolongation of PFS by CAB in prostate cancer patients without significant risk factors. It would possible to decide the type of the first‐line ADT according to the prostate cancer risk.

## 1 INTRODUCTION

Androgen deprivation therapy (ADT) occupies an important position in the treatment of prostate cancer and is used as a main treatment for prostate cancer of all stages.^1^, ^2^, ^3^ In general, ADT is achieved by surgery, drug‐based monotherapy, or combined androgen blockade (CAB).^3^, ^4^, ^5^, ^6^

ADT results in a marked decrease in the prostate‐specific antigen (PSA) level and shows a good tumor reduction effect in most cases.^7^ However, the PSA may be found to gradually increase even when the testosterone value is at a castration level. Such biochemical relapse is called castration‐resistant prostate cancer (CRPC).^8^, ^9^, ^10^ There are several definitions of CRPC, but the current guidelines do not take into consideration use of anti‐androgens up to CRPC.^8^, ^9^, ^10^

For this reason, it is necessary to recognize two important points concerning CRPC. First, CRPC patients have various treatment histories. Thus, at the time of CRPC determination, patients may have different tumor biological properties. Second, the relationship between the type of ADT and the period from treatment start to CRPC (time to CRPC) has not been sufficiently studied. If there were a big difference in the time to CRPC depending on the type of ADT, then the type of ADT would be an important factor when considering the entire course of treatment of prostate cancer.

These matters are of little concern if endocrine therapy is being carried out by either method alone. Looking at the positioning of ADT as the first‐line main treatment in Western guidelines, ADT itself is not listed as a treatment option for localized cancer, and they go no further than indicating the possibility that CAB is slightly useful for advanced cancer.^9^, ^10^ However, in reality, according to a paper based on an observational study, CAB was selected for 46% of prostate cancer patients in the US CaPSURE registry and for 59% to 74% in Japanese studies.^2^, ^3^, ^5^ Formerly, the overall survival (OS) was employed as the primary endpoint in studies of the usefulness of CAB that were conducted before the 2000s, when excellent CRPC therapeutic drugs came into use. However, considering that the survival period after CRPC has been significantly extended by the use of new drugs,^11^, ^12^, ^13^, ^14^, ^15^ we have reached a point where it is necessary to focus on the treatments administered up to CRPC and revisit the question of the optimal ADT method.

This study investigated the optimal method for ADT with the aim of solving some of the current problems confronting treatment of prostate cancer.

## 2 PATIENTS AND METHODS

### 2.1 Patients

We used a data set from a Japanese multi‐institutional registry prepared by the Japan Study Group of Prostate Cancer consisting of 17 388 patients (almost all patients were Japanese) who started ADT as the first‐line main treatment between 1 January 2001 and 31 December 2003. This database includes the follow‐up patient status up to 31 September 2014, when the data were locked. Approval of data collection was obtained by the institutional review board. All the data were anonymized so as to protect the identities of subjects. Among all registered patients, 1009 patients treated with anti‐androgen monotherapy and another 2605 patients who started CAB with an anti‐androgen other than bicalutamide and flutamide were also excluded because the drugs were selected for unusual conditions or are rarely used nowadays.^2^, ^9^ Thus, the remaining 13 774 patients were the subjects of this study.

### 2.2 Methods

The characteristics of tumors were determined by each physician in the usual clinical practice framework, basically according to the Japanese guideline.^16^ Then, the clinical TNM stages were determined according to the UICC 5th edition,^17^ and Gleason scores were determined according to the 1977 version.^18^ Prostate cancer risks were determined by the J‐CAPRA scoring system, consisting of the initial PSA, Gleason score, and TNM category.^19^ The type of primary ADT was classified as monotherapy (surgical or medical castration) or CAB (surgical or medical castration plus a nonsteroidal anti‐androgen), as described in our earlier paper.^5^ The data set used was derived from the real world, so the treatment was not randomly selected, and had been decided by the patient and/or physician.

### 2.3 Analysis of survival

Diagnosis of progression was performed by each physician, basically depending on the aforementioned guideline.^16^ This guideline defined two types of progression: PSA progression and clinical progression. PSA progression was defined as three consecutive PSA re‐rises following PSA decrease by ADT under a low testosterone level in response to ADT. The first date of consecutive PSA rise was defined as the PSA progression date. Clinical progression was defined as regrowth of a tumor or development of a new lesion. For analysis of the progression‐free survival (PFS), either type of progression, or death, caused by the prostate cancer was defined as an event. For analysis of the OS, death due to any cause was considered an event. For analysis of the cancer‐specific survival (CSS), only death due to the prostate cancer was considered an event. Patients without any event were censored at the last follow‐up visit. The number of days from initial hormonal manipulation to the earliest date of these events or the censored date was calculated for analyses. The median follow‐up duration estimated by the reverse Kaplan‐Meier method was 3.7 years.

### 2.4 Statistical analysis

Correlations between the clinicopathological background and the type of ADT were tested using the Cochran‐Armitage trend test and a logistic regression model. The survival rate was estimated by the Kaplan‐Meier method, and survival differences between different groups were examined by the log‐rank test. The hazard ratio of each variable was calculated using Cox's proportional hazard model. The propensity score, that is, the probability of CAB use, was calculated using a logistic regression model in which potential confounders, that is, age, TNM category, initial PSA value, and Gleason score, were used as independent variables, and the type of ADT was used as the dependent variable. One‐to‐one propensity score‐matched pairs were selected from the two treatment groups by nearest neighbor matching. An open‐source software, R version 3.3.3,^20^ was used for statistical analyses. All tests were two‐sided, and a *P*‐value of <0.05 was considered statistically significant.

## 3 RESULTS

### 3.1 Background characteristics

Table 1 shows the patient characteristics. The median patient age was 75 years (interquartile range (IQR), 71‐80). A total of 6225 (45.2%) patients had organ‐confined disease, while 2514 (18.3%) and 4359 (31.6%) patients had clinical stage III and stage IV disease, respectively.

**Table 1:** Background characteristics according to the type of androgen deprivation therapy

Characteristic	Total (no.)	Monotherapy (no.)	CAB (no.)	CAB% (%)	*P*‐value
Total	13 774	5395	8379	60.8	
Age at diagnosis (y)					<0.001
70	3233	1029	2204	68.2	
>70 and ≤75	3850	1408	2442	63.4	
>75 and ≤80	3711	1599	2112	56.9	
>80	2980	1359	1621	54.4	
PSA at diagnosis (ng/mL)					<0.001
≤20	6200	2858	3342	53.9	
>20 and ≤100	4290	1579	2711	63.2	
>100 and ≤500	2017	612	1405	69.7	
>500	1267	346	921	72.7	
Gleason score					<0.001
2 to 6	4212	1896	2316	55.0	
7	3497	1451	2046	58.5	
8 to 10	4287	1405	2882	67.2	
Not available	1778	643	1135		
T category					<0.001
T1	2899	1387	1512	52.2	
T2	4493	1868	2625	58.4	
T3	5038	1714	3324	66.0	
T4	1291	399	892	69.1	
Not available	53	27	26		
N category					<0.001
N0	11 141	4552	6589	59.1	
N1	1926	532	1394	72.4	
Not available	707	311	396		
M category					<0.001
M0	9749	4159	5590	57.3	
M1	3525	1013	2512	71.3	
Not available	500	223	277		
Clinical stage					<0.001
I to II	6225	2844	3381	54.3	
III	2514	963	1551	61.7	
IV	4359	1285	3074	70.5	
Not available	676	303	373		
J‐CAPRA risk category					<0.001
Low risk	5156	2454	2702	52.4	
Intermediate risk	4129	1442	2687	65.1	
High risk	1924	517	1407	73.1	
Not available	2565	982	1583		

CAB, combined androgen blockade; CI, confidence interval.

With respect to the type of ADT, monotherapy and CAB were used in 5395 (39.2%) and 8379 (60.8%) patients, respectively. The type of the first castration was medical castration in 12 598 patients (91.4%; leuprolide and goserelin in 8577 and 4021 patients, respectively) and surgical castration in 1176 patients (8.5%). The first anti‐androgen used in the CAB group was bicalutamide 80 mg in 7202 patients (86.0%) and flutamide in 1177 patients (14.0%).

### 3.2 Type of androgen deprivation therapy according to background characteristics

The proportion of patients treated by CAB became significantly higher as patient age became significantly younger and the tumor risk became higher (*P* < 0.001, Table 1). Multivariate analysis using a logistic regression model revealed that younger age, higher PSA value at diagnosis, higher Gleason score, and nodal or distant metastasis were positively associated with CAB use (Table 2).

**Table 2:** Background characteristics accounting for the type of androgen deprivation therapy

Characteristic	Category	Odds ratio for CAB use					
		Univariate			Multivariate		
		Odds ratio	(95% CI)	*P*‐value	Odds ratio	(95% CI)	*P*‐value
Age at diagnosis (y)	≤70	1.8	(1.6‐2.0)	<0.001	1.7	(1.5‐1.9)	<0.001
	>70 and ≤75	1.5	(1.3‐1.6)	<0.001	1.5	(1.3‐1.7)	<0.001
	>75 and ≤80	1.1	(1.0‐1.2)	0.039	1.2	(1.1‐1.3)	0.004
	>80	Ref			Ref		
PSA at diagnosis (ng/mL)	≤20	Ref			Ref		
	>20 and ≤100	1.5	(1.4‐1.6)	<0.001	1.3	(1.2‐1.5)	<0.001
	>100 and ≤500	2.0	(1.8‐2.2)	<0.001	1.5	(1.3‐1.7)	<0.001
	>500	2.3	(2.0‐2.6)	<0.001	1.4	(1.1‐1.7)	0.001
Gleason score	2 to 6	Ref			Ref		
	7	1.2	(1.1‐1.3)	0.002	1.0	(0.9‐1.1)	0.714
	8 to 10	1.7	(1.5‐1.8)	<0.001	1.3	(1.2‐1.4)	<0.001
T category	T1	Ref			Ref		
	T2	1.3	(1.2‐1.4)	<0.001	1.1	(1.0‐1.2)	0.066
	T3	1.8	(1.6‐2.0)	<0.001	1.1	(1.0‐1.3)	0.054
	T4	2.1	(1.8‐2.4)	<0.001	1.0	(0.8‐1.2)	0.879
N category	N0	Ref			Ref		
	N1	1.8	(1.6‐2.0)	<0.001	1.2	(1.0‐1.3)	0.043
M category	M0	Ref			Ref		
	M1	1.8	(1.7‐2.0)	<0.001	1.4	(1.3‐1.6)	<0.001
Clinical stage	I to II	Ref					
	III	1.4	(1.2‐1.5)	<0.001			
	IV	2.0	(1.9‐2.2)	<0.001			
J‐CAPRA risk category	Low risk	Ref					
	Intermediate risk	1.7	(1.6‐1.8)	<0.001			
	High risk	2.5	(2.2‐2.8)	<0.001			

CAB, combined androgen blockade; CI, confidence interval; Ref, reference.

### 3.3 Survival rates according to clinical stages

The PFS rate was 58.3% (95% confidence interval (CI), 57.3% to 59.5%) at 5 years (75.9%, 63.2%, and 30.4% for clinical stage I to II, III, and IV, respectively; *P* < 0.001). The CSS rate was 87.7% (87.0% to 88.5%) at 5 years (75.9%, 63.2%, and 30.4% for clinical stage I to II, III, and IV, respectively; *P* < 0.001). The OS rate was 76.6% (75.7% to 77.6%) at 5 years (75.9%, 63.2%, and 30.4% for clinical stage I to II, III, and IV, respectively; *P* < 0.001).

### 3.4 Survival rates according to the type of androgen deprivation therapy

#### 3.4.1 Propensity score matching and survival in matched patients

**Figure 1:**
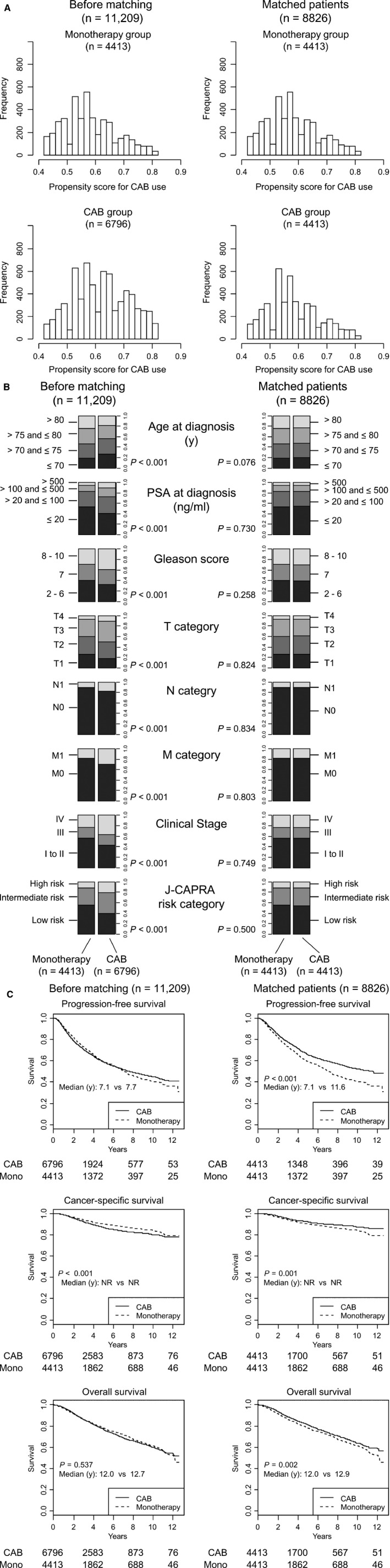
Background characteristics and survival rates of patients treated by monotherapy or combined androgen blockade before and after propensity score matching. A, Distribution of propensity scores for CAB use in monotherapy and CAB groups before and after propensity score matching. After matching (right), the distribution of the propensity scores in each treatment group became similar. B, Background characteristics in monotherapy and CAB groups before and after propensity score matching. Before matching, patients treated with CAB were characterized by being significantly younger and having higher risk factors (left). However, after matching, there were no differences in background characteristics between the two treatment groups (right). C, Survival rates before and after propensity score matching. After matching, the PFS rate was significantly higher in favor of CAB (right). Numbers below the graph represent patient numbers at risk. CAB, combined androgen blockade; NR, not reached to median

Due to the aforementioned results that younger patients and patients at higher risk tended to receive CAB more frequently (Tables 1 and 2), we concluded that the two treatment groups were not comparable and that imbalanced background variables act as confounding factors if we simply compare the survival between the two treatment groups. We therefore performed propensity score matching to achieve comparability. A total of 11 209 patients with no missing data included 8826 matched patients (Figure 1A). The backgrounds of the matched patients were well balanced (Figure 1B). Before matching, survival was similar (PFS and OS) or significantly better (CSS) in the monotherapy group. However, after controlling the backgrounds, the PFS rate was significantly higher in the CAB group compared with the monotherapy group (65.6% and 59.6% at 5 years, respectively; time to progression, 11.6 vs 7.1 years; hazard ratio in the CAB group, 0.78 (95% CI, 0.72 to 0.84, *P* < 0.001) (Figure 1C). Likewise, CSS and OS were significantly higher in the CAB group (Figure 1C).

#### 3.4.2 Subgroup analysis

**Figure 2:**
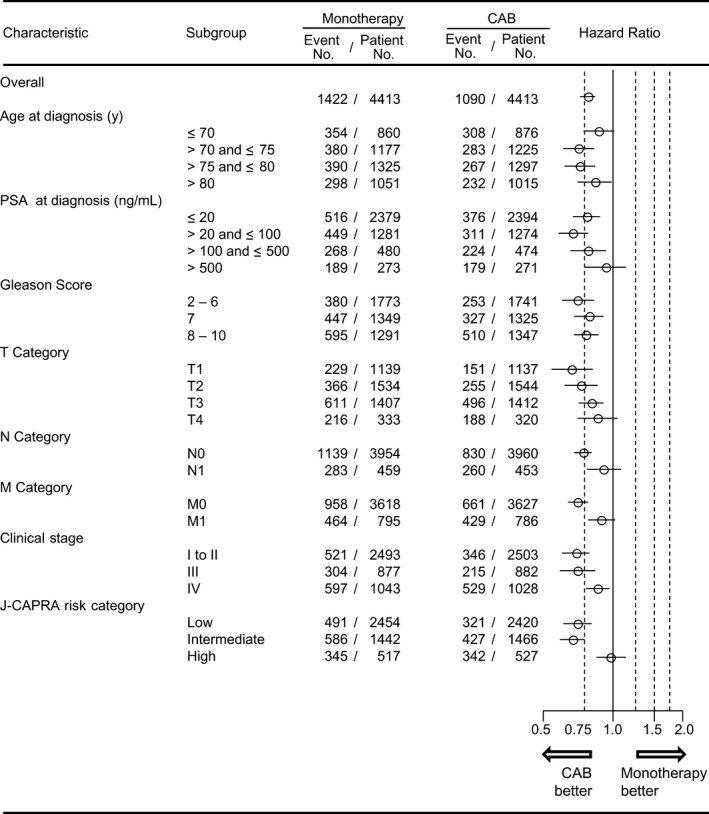
Progression‐free survival (PFS) according to the type of androgen deprivation therapy within subgroups. A higher PFS rate was demonstrated in the CAB group in all risk subgroups except for extremely high‐risk categories. Horizontal lines in the hazard ratio represent 95% confidence interval. CAB, combined androgen blockade; NR, not reached to median; CI, confidence interval; PFS, progression‐free survival

We further compared the PFS in the two treatment groups within each background strata. As shown in Figure 2, the PFS rate was significantly higher in the CAB group compared with the monotherapy group in all risk subgroups except for those with the highest risk factors (PSA exceeding 500 ng/mL, T4, N1 or M1 disease, and J‐CAPRA high‐risk category).

## 4 DISCUSSION

The data set we used was derived from the real world, and selection of the type of ADT was not randomized. In fact, in the data used, as in an independent report,^2^ CAB was more frequently selected for cases with high‐risk factors and young cases. Meta‐analyses showed that the OAB was better with CAB by nonsteroidal anti‐androgen than with monotherapy during the time period from 2001 through 2003, when the patients were registered in the database.^21^ There is a possibility that this may have influenced selection of the type of ADT. This type of imbalance is widely known as “confounding by indication.”^22^, ^23^ We used propensity score matching to control for confounding factors in this study.^24^ As a result, the CAB showed significantly better results in terms of each of the PFS, CSS, and OS. Also, in subgroup analysis, the CAB group showed a better PFS in most subgroups, except for some subgroups with high‐risk factors.

Our present results suggest that it is possible to select the type of ADT according to the risk of prostate cancer. In J‐CAPRA low‐risk cases, the CAB group showed a significantly better PFS, and it can be thought that CAB was a reasonable choice. However, low‐risk cases showed a good PFS in excess of several years even with monotherapy, so monotherapy may be adequate for some patients. In intermediate‐risk cases, the CAB group also showed a time to progression that was 43.9% longer than in the monotherapy group, so CAB can again be considered to have been a reasonable choice. Today, CRPC therapeutic drugs are 10 to 20 times more expensive than the conventional nonsteroidal anti‐androgens, so if you extend the time to progression by 10% to 5%, respectively, medical expenses at the time of CRPC in the CAB group can be reduced. Choosing CAB can thus be considered reasonable in terms of the cost, as well. On the other hand, in J‐CAPRA high‐risk cases, for whom ADT is generally indicated, CAB did not improve the PFS. Therefore, magnitude of clinical significance of ADT including CAB might be limited in this risk group, and a stronger treatment is required. Recent studies tested docetaxel or abiraterone in combination with first‐line ADT for advanced prostate cancer and showed prolongation of both the OS and the time to progression in the combination group.^25^, ^26^, ^27^ Therefore, as NCCN Guidelines mention,^10^ combined use of docetaxel or abiraterone from the beginning of ADT should be considered for these patients.

The problem exposed by our present study is the ambiguity of the definition of CRPC. Currently, the definition of CRPC in various guidelines is re‐elevation of the PSA value even though the testosterone level is low, while the use or nonuse of anti‐androgens is not considered.^8^, ^9^, ^10^ However, this study's finding that the time to progression varies greatly depending on the type of ADT is important. This is because the biological properties of the tumors differ between the CAB group and the monotherapy group even though the cases were uniformly judged to be CRPC. As an example, in the STRIVE trial comparing enzalutamide and bicalutamide 50 mg for nonmetastatic and metastatic CRPC patients, bicalutamide caused the PSA to decline in one‐third of CRPC patients who had been naïve to bicalutamide.^28^ Based on that finding, it can be thought that we need to modify the definition of CRPC by incorporating the history of use of anti‐androgens, or, in the absence of such modification, we need to clarify the use or nonuse of anti‐androgens and then conduct various investigations. Accordingly, if we were to evaluate the “time from ADT to CRPC” and the “time from CRPC to death” individually, then we would be able to perform more detailed analyses of prostate cancer treatments' contents and the treatment course. We think this approach is essential for devising a sequential strategy for prostate cancer drug therapy for the future.

This study has a number of limitations. One is that the TNM and Gleason score classification methods used were different from the current versions.^8^, ^16^ A second is that the main endpoint, that is, progression, was judged by individual urologists based on diagnostic criteria that were current at the time. A third is that our database did not include detailed information concerning use of anti‐androgens midway through the treatment course. A fourth is that almost all the patients in this data set were Japanese, so that the findings obtained in the present study might be specific to Japanese and might be different in other ethnic groups. Moreover, the data used for the study were derived from the real world, so the type of ADT was not randomized; however, we attempted to eliminate confounding factors by propensity score matching. On the other hand, our study employed data for more patients than in similar studies published to date. Also, the study cohort included prostate cancer patients of all stages, not just advanced cancer, and the patients were close to actual clinical cases. We think these advantages provide sufficient value, even in view of the above limitations.

In conclusion, this study used propensity score matching and found that the PFS, CSS, and OS were better in the CAB group compared with the monotherapy group. In particular, the difference in the PFS due to the type of ADT type was remarkable, and the utility of CAB was widely shown, except for in some high‐risk subgroups. As the prognosis after progression to CRPC has improved in recent years due to various new drugs, it is now time to reevaluate our treatment strategies up to CRPC. The findings generated in this study show the feasibility of selection of the type of the first‐line ADT (castration monotherapy vs castration plus nonsteroidal anti‐androgen vs castration plus new drugs) according to the risk and also show that the type of ADT is, in itself, an important clinical factor. Therefore, it might be worthwhile to reconsider the definition of CRPC based on the presence or absence of anti‐androgen use up to progression.

## CONFLICT OF INTEREST

None declared.
